# A Chinese medicine warm compress (Wen Jing Zhi Tong Fang), combined with WHO 3-step analgesic ladder treatment for cancer pain relief: A comparative randomized trial

**DOI:** 10.1097/MD.0000000000009965

**Published:** 2018-03-16

**Authors:** Peiling Cai, Liuning Li, Hongxi Hong, Liwen Zhang, Chunxia He, Xiaoshu Chai, Bai Liu, Zhijian Chen

**Affiliations:** a The Second Medical School, University of Guangzhou Traditional Chinese Medicine; b Department of Medical Oncology, Guangdong Provincial Hospital of Chinese Medicine, The Second Clinical Medical College, University of Guangzhou Traditional Chinese Medicine, Guangzhou, Guangdong Province, China.

**Keywords:** back meridians, cancer-related pain, Chinese medicine, warm compress

## Abstract

**Introduction:**

This study aimed to assess the effectiveness of Chinese medicine warm compress (CMWC) on back meridians in relieving cancer pain, reducing adjuvant analgesic doses and adverse reactions, and improving the quality of life (QOL).

**Methods:**

A total of 62 patients (age range 39–82 years) diagnosed with a malignant tumor and suffering from cancer-related pain were randomly divided into a treatment group (group A) and a control group (group B) (n = 31 for each). The patients in both groups were administered appropriate drugs for 2 cycles of 7-day treatments according to the World Health Organization (WHO) 3-step ladder for cancer pain relief in adults. In addition, a CMWC was given to patients in group A. Pain relief was assessed using the visual analogue scale (VAS) at various time points before and after interventions in each group. Alteration of analgesic doses, adverse reactions, performance status (PS), and QOL were evaluated and any differences between groups A and B evaluated.

**Results:**

VAS scores at various time points after treatment were significantly decreased compared with the baseline level in group A. Overall response rate was significantly improved in group A compared with group B (70.97% vs 29.03%, *P* < .001). Significant differences in clinical pain relief efficacy in various locations were found in group A after treatment vs before treatment (*P* < .05). Adjuvant analgesic doses were significantly changed in the control group compared to the treatment group after 1 cycle of 7-day treatment (22.58% vs 12.90%, *P* = .023). QOL were improved more in group A than in group B (3.00 ± 4.23 vs −2.06 ± 2.38, *P* < .001). Significantly reduced adverse reactions were observed after treatment of group A compared with group B in terms of the overall incidence (3.23% vs 80.65%, *P* < .05) or incidence of constipation (3.23% vs 77.42%, *P* < .05).

**Conclusions:**

The application of CMWC on back meridians combined with WHO 3-step analgesic ladder treatment was effective in relieving cancer-related pain with reduced doses, less adverse reactions, and improved QOL.

## 1 Introduction

Cancer is the leading cause of death in China. In 2015, it was estimated that about 4,292,000 patients with invasive cancer would be newly diagnosed and about 2,814,000 were expected to die.^[1]^ Although advances in screening and treatments have extended the lifespan of patients with cancer, cancer-related pain remains a problematic symptom in the advanced stages,^[2]^ which adversely affects their quality of life.^[3,4]^ Cancer pain is complex with numerous mechanisms operating that include inflammatory, neuropathic, ischemic and compressive changes at multiple sites in the body.^[5]^ Pain can be caused by inflammatory mediators^[6]^ (such as endothelin-1, prostaglandin E, and tumor necrosis factor alpha), which are released from tumor-associated macrophages, mast cells, neutrophils and T lymphocytes. In addition, cancer patients often have disorders of the blood microcirculation and pain can be triggered by vasodilatation, induced by free chemicals and local blood stasis.^[7]^ The endothelin-B receptor (ET_B_R) is believed to mediate inflammatory pain and vasodilatation.^[8,9]^ The characteristics of cancer pain vary depending on the duration, location, and type of cancer. Even with the broad availability of analgesics, more than 50% of cancer patients were undertreated for the relief of moderate and severe pain symptoms.^[2,10]^

One common approach to cancer pain management employs the guideline of the 3-step pain ladder introduced by the World Health Organization (WHO),^[11]^ which suggested that prompt oral administration of nonopioid analgesics with or without adjuvants, low-potency opioids plus nonopioid analgesics with/without adjuvants, and potent opioids plus nonopioid analgesics with/without adjuvants should be used to treat mild pain (Step 1), medium pain (Step 2) and intense pain (Step 3), respectively. Long-term use of analgesics for cancer pain should consider concerns about side effects. Nonopioid analgesics may produce gastrointestinal bleeding, kidney and liver dysfunction, cardiovascular toxicity and allergic reactions. The adverse effects of opioids include constipation, itchiness, urinary retention, nausea, and dizziness. Serious consequences may be coma and respiratory depression. In addition, opioid analgesics may lead to addiction or acute toxicity, affect physiological and psychological well-being, cause social phobia and also a reduced QOL.^[12,13]^

Traditional Chinese medicine (TCM) has a long history in the treatment malignancies and believes that impediment of collaterals and meridians is the origin of pathogenic of cancer pain. Long-term stagnation of vital energy (*qi*) and stasis of blood are thought to result in “pain due to impediment” and “pain due to lack of nourishment.” TCM treatment for cancer pain involves the use of traditional Chinese medication to restore the balance of the internal environment by reinforcing and adjusting *qi to* circulate through meridians, which connect various internal organs and functions.^[14]^ Studies have shown that the application of Chinese herbal medicine (CHM) and acupuncture increase the peripheral release of endogenous analgesic agents, reduce pain mediator secretion and induce central nervous system (CNS) analgesia.^[15–18]^ These studies demonstrated that the use of TCM to treat pain triggered by cancer is effective and economical and furthermore produces fewer side effects.^[19]^

According to the statement of Wu Shiji, a great master of external treatment in the Qing Dynasty, the external treatment is the same as the endotherapy in theory; CHM for external treatment is also the same as endotherapy. CHM for external treatment can be absorbed through the skin, infiltrate the skin and reach the target meridians for its actions. Wen Jing Zhi Tong Fang, a CMWC, is a CHM and composed of *evodia rutaecarpa* (*Wuzhuyu*), *semen sinapis* (*Baijiezi*), *ephedra sinica* (*Mahuang*), and *asarum sieboldii* (*Xixin*). It is designed for cancer pain relief through dredging healthy *qi* to warm and activated blood and by promoting the circulation through the back meridians, which is considered to be the source of *yang qi* (healthy *qi*) and have broad connections with the viscera. In the present study, we investigated the efficacy and safety of a CMWC on cancer pain treatment combined with the WHO 3-step analgesic ladder treatment.

## 2 Methods

### 2.1 Patients

The current study was carried out in the Department of Medical Oncology of the Guangdong Provincial Hospital of Chinese Medicine between December 2016 and January 2017. Patients were enrolled if they were diagnosed with a malignant tumor, suffered from mild to severe cancer-related pain and agreed to take part in the study. Patients were excluded if they had grave skin lesions, drug allergy, dysfunction of vital organs (heart, liver, and kidneys), mental disorders, severe idiopathic disease, or bad compliance. As a result, a total of 62 patients (32 men and 30 women) aged 39 to 82 years were recruited and randomly allocated into the treatment group (group A) or the control group (group B) (n = 31 for each group). The WHO 3-step analgesic ladder for the relief of cancer pain with and without CMWC, named as “Wen Jing Zhi Tong Fang” in Chinese, were given to patients in the treatment and control groups, respectively. Written informed consent was obtained from every patient before enrolment and approval for the study was granted by the Ethics Committee of Guangdong Provincial Hospital of Chinese Medicine (no. B2016-131-01).

### 2.2 Study procedure

The number of samples to study the effect of Chinese medicine in cancer pain patients has been estimated to be at least 30 by preliminary treatments. The participants were randomly divided into the research group and the control group, using unbiased systematic sampling. The patients in the 2 groups were tested individually before intervention and 12-, 24-, 48-, and 72 hours, as well as 7- and 14 days after intervention initiation and the cancer pain assessment, analgesic doses the types of drugs administered, adverse events, performance status as well as QOL for each participant was recorded. Comparison of before versus 14-days after treatment initiation between the treatment and control groups served as data for clinical efficacy of treatment and safety evaluations.

### 2.3 Treatments

All participants in both the treatment and control groups were given drugs for cancer pain relief for 2 cycles (7-day per cycle) according to the following guideline of the WHO 3-step analgesic ladder: step 1 for minor pain with a nonopioid analgesic (celecoxib, ibuprofen) with/without an adjuvant analgesic; step 2 for moderate pain with a low-potency opioid (codeine, dihydrocodeine, tramadol) with or without a nonopioid or adjuvant drug; step 3 for severe pain with a strong opioid (such as morphine, fentanyl, oxymorphone, or oxycodone with/without a nonopioid or adjuvant drug.

In addition to the WHO 3-step analgesic ladder medication regime, patients in the treatment group were also given a CMWC, ‘Wen Jing Zhi Tong Fang’, which was composed of *Evodia rutaecarpa* (*Wuzhuyu*, 120 g), *Semen sinapis* (*Baijiezi*, 120 g), *Ephedra sinica* (*Mahuang*, 30 g) and *Asarum sieboldii* (*Xixin*, 30 g). The herbs were packed in a warm packet of size 40 × 20 cm^2^, heated and deposited on the back meridians of patients (from T1 to T12 vertebrae; *Du*, *Back Shu*, *Jiaji* point) in the supine position for approximately 30 minutes, once daily for 2 weeks.

### 2.4 Measurement and assessment

#### 2.4.1 Measurement of pain intensity

The pain intensity was assessed using the visual analogue scale (VAS)^[20]^ and numeric rating scale (NRS)^[21]^ VAS: The patients marked their pain on a 100 mm line, with 0 mm at the left side and 100 mm at the right side, indicating no pain and the most excruciating pain, respectively. The intensity of the pain score was calculated by measuring the distance in mm between the “no pain” mark and the patient's mark, producing scores ranging from 0 to 100. VAS scores have been recommended as the following grades (in mm): no pain (0–4), mild pain (5–44), moderate pain (45–74), and severe pain (75–100). NRS: The patients rated their pain from 0 to 10 and the pain level was divided into the following 4 grades: 0 for no pain (grade 0), 1 to 3 for mild pain (grade I, background pain having no significant effects on the activities of daily living (ADLs), 4 to 6 for moderate pain (grade II, significantly interfering with ADLs) and 7 to 10 for excruciating pain (grade III, marked interference with the ADLs).

#### 2.4.2 Evaluation of clinical efficacy

Pain relief was compared before versus 14 days after treatment initiation by using VAS scores to determine the clinical efficacy of treatment. The clinical efficacy of treatment in each group was described as follows: Complete relief (CR): no pain experienced (VAS = 0); partial relief (PR): the pain was significantly alleviated (>50%), sleep disturbance was not detected and the patients lived normally; minimal relief (MR): the pain was obvious but significantly reduced (30%–50%) and sleep disturbance was detected; no effect (NR): the pain level was unchanged (<30%) or was more serious than before. PR and CR were taken as effective responses to the treatment of cancer pain (ORR: overall response rate).

#### 2.4.3 Assessment of quality of life

Performance status and quality of life (QOL) was measured before and 14 days after treatment initiation for each participant. PS was used to quantify patients’ general well-being and ADLs. The Zubrod score or Eastern Cooperative Oncology Group (ECOG) score was scored from 0 to 5, with 0 denoting normal health and a score of 5 death.^[22]^ In addition, QOL was measured by a health form survey comprised of 12 items (SF-12) that measured 8 health domains, including general well-being, physical functions, physical role, body pain, vitality, social functioning, emotional role and the mental state of the patient.^[23]^ The scores of QOL with a maximum at 60 points were divided and interpreted as the following: <20 very poor; 21 to 30 poor; 31 to 40 fair; 41 to 50 good; and 51 to 60 very good.

#### 2.4.4 Analgesic doses

Analgesic doses and the types of drugs administered were compared between 7 and 14 days after treatment initiation with before treatment in each group and recorded as follows: no change; dose reduced; dose increased; medication changed.

#### 2.4.5 Safety evaluation

The safety evaluation was based on adverse events, which were recorded during the treatment with analgesics with or without the CMWC. The degrees of constipation was rated as grade 1 to 5^[24]^ with a detailed clinical description as follows: grade 1, intermittent or occasional symptoms, occasional use of laxatives or stool softeners, modification of diet, or enema use; grade 2, persistent symptoms with routine use of enemas or laxatives, inhibition of the ADLs; grade 3, constipation with manual evacuation required, inhibition of self-caring ADLs; grade 4, life-threatening circumstances requiring urgent interventions; and grade 5, the patient died.

### 2.5 Statistical analyses

All statistical analyses were carried out using SPSS ver. 17.0 (SPSS, IL). The continuous and categorical data are shown as the mean ± standard deviation and percentages, respectively. The chi-squared analysis was applied to compare clinical efficacy between the treatment and control groups. Comparison of changes in analgesic doses between the treatment and control groups were measured using a Fisher exact test. A Wilcoxon rank-sum test was employed to compare PS and the incidence of constipation between the 2 groups. A paired repeated-measures analysis of variance (ANOVA) and a *t* test were used for comparative analysis of VAS scores and QOL before and after treatment between the 2 groups, respectively. A *P* < .05 was considered to be statistically significant.

## 3 Results

### 3.1 Characteristics of patients

A total of 62 (32 men and 30 women) were eligible for the study. The mean age of patients was 58.85 ± 11.38 years (range 39–82). The patients pain was located in various regions including the chest and rib (n = 6), abdomen (n = 15), lower back (n = 3), chest and back (n = 9), upper and lower back (n = 3), upper back (n = 3), abdominal and lower back (n = 8) and >2 locations (n = 15). There were 19, 22 and 21 patients who suffered from mild, moderate and severe pain, respectively. The patients were divided at random into 2 groups (31 patients each for the treatment and control groups, respectively) using a stratified sampling method and according to the registration sequence. No significant differences were found between the 2 study groups with regard to sex, age, pain location or its intensity (*P* > .05) (Table 1).

**Table 1:**
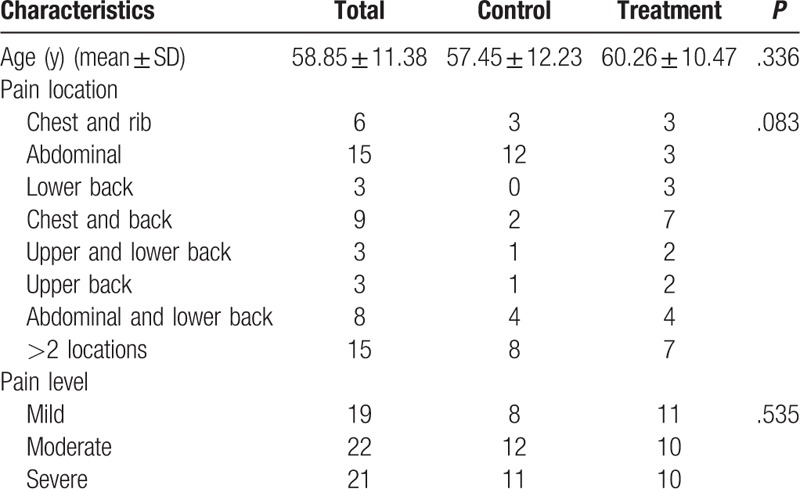
Characteristic features of patients in the treatment and control group (n).

### 3.2 Evaluation of clinical efficacy

**Figure 1:**
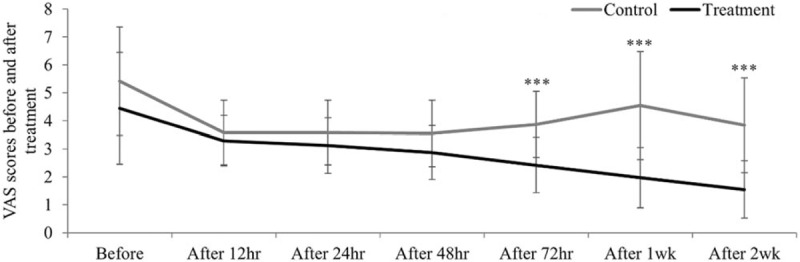
VAS scores before and after treatment at several time points in both the groups: Measured by repeated-measures ANOVA (*F* = 21.680, *P* = .000 < .05). VAS = visual analogue scale.

The VAS scores obviously decreased in the treatment group and a statistically significant difference was found (*P* = .000), especially at 12-, 24-, 48- and 72 hours, 7 days and 14 days after treatment initiation between the treatment and control groups (*P* = .454, .317, .202, .000, .000, .001, respectively; Fig. 1). The ORR (CR + PR) was 29.03% and 70.97% for the control and treatment groups, respectively, and the difference was statistically significant (*P* = .001, Table 2).

**Table 2:**
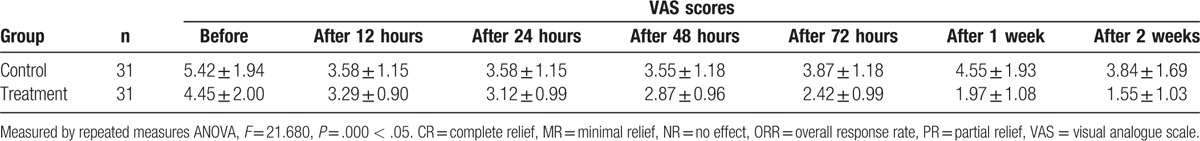
Comparison of VAS scores before and after treatment at several time points.

**Figure 2:**
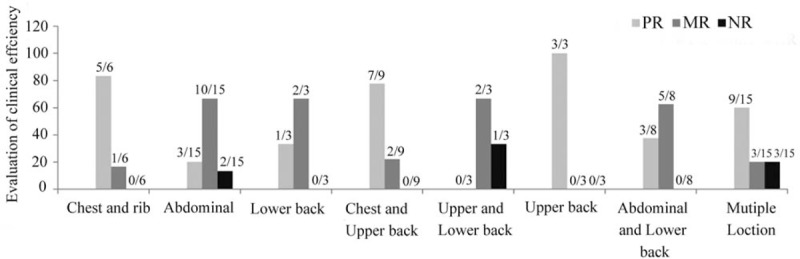
Comparison of clinical efficiency for several pain locations: Comparison of clinical efficiency in several pain locations measured by the Kruskal–Wallis test (*χ*^2^ = 16.812, *P* = .019 < .05).

Next, we compared the clinical efficacy of pain relief based on different pain locations. We found no complete remission for patients in all different pain locations; upper back pain patients received 100% PR, followed by 83.3% PR of chest and rib pain and 77.8% PR of chest and upper back pain. The ORR of pain relief was 100%, 83.33%, 77.78%, 60%, 37.5%, and 33.33% for locations in the upper back, chest and rib, chest and upper back, multiple locations, abdominal regions, and the lower back, respectively; the differences were statistically significant (*P* = .019, *χ*^2^ = 16.812) (Table 3, Fig. 2). These data suggested that the treatment group had a significantly higher clinical efficacy in terms of pain relief compared with the control group.

**Table 3:**
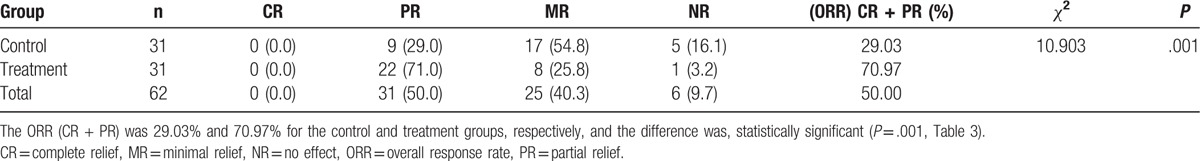
Comparison of the efficacy of pain relief in the 2 groups.

### 3.3 Alteration of analgesic doses and type after treatment

The changes of analgesic doses and medication between control and treatment group 7- and 14-day after treatment initiation were compared and are summarized in Table 4. Analgesic doses were increased in 9 (29.03%) and 2 (6.45%) patients respectively and medication changed in 7 (22.58%) and 4 (12.90%) patients in the control and treatment groups after 7-day post treatment start. Nobody in the control group compared with 1 patient (3.22%) in the treatment group required a reduced analgesic dose. The difference between the control and treatment groups 7 days posttreatment initiation was statistically significant (*P* = .023), suggesting less medication adjustment in the treatment group compared with the control group. However, no significant difference of alterations in analgesic doses and medication were found between the control and treatment groups after 14 days treatment (*P* = .287).

**Table 4:**
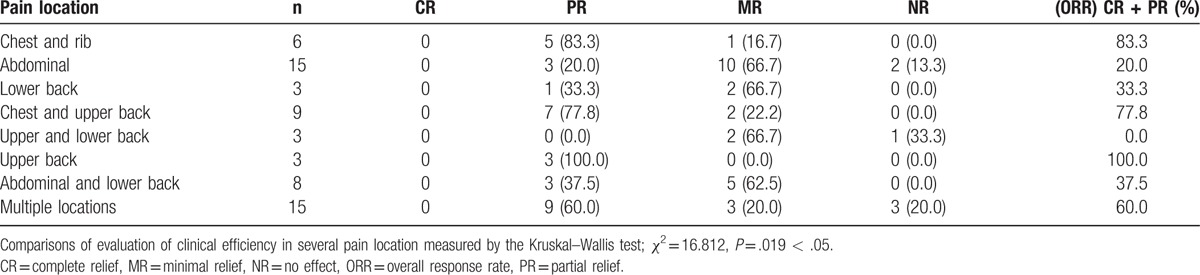
Comparison of evaluations of clinical efficiency for several pain locations (n, %).

### 3.4 Quality of life

Comparison of the PS scores 14-day after treatment initiation in both groups was measured and the results are shown in Table 5. After 14-day treatment, the PS score increased in 5 cases (5/31) in the control group, including 1, 2 and 2 patients increased 1 point from the baseline PS score at 1, 2, and 3, respectively. In contrast, a decline in the PS score was observed in 9 cases (9/31) in the treatment group, including 4 and 4 patients reduced 1 point from the baseline PS score at 2 and 3, respectively, and 1 patient reduced 2 points from the baseline PS score of 3. The difference of PS score between the control and treatment group after 14-day treatment was statistically significant (*P* = .000).

**Table 5:**
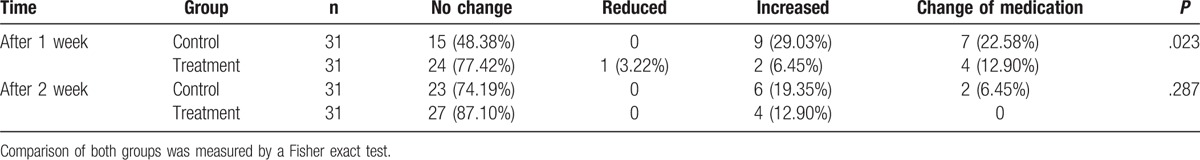
Comparison of analgesic doses after treatment (n, %).

As summarized in Table 6, QOL score increased −2.06 ± 2.38 and 3.00 ± 4.23 points in control and treatment group 14-day posttreatment initiation, respectively, compared with that before treatment. The difference of QOL score was statistically significant (*P* = .000).

**Table 6:**
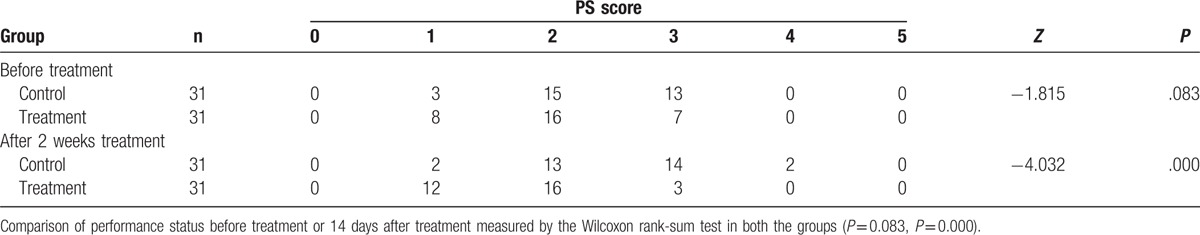
Comparison of performance status before treatment and after 2 weeks treatment (n).

The results suggested that the use of integrated modern medicine and CMWC on back meridians improved PS and QOL.

### 3.5 Safety evaluation

Adverse events (AEs) were recorded before and during the period of analgesic treatment. By 14-day treatment, there was only events of mild constipation reported in the treatment group, in contrast to events of mild-to-severe constipation, nausea and vomiting, urinary retention and other symptoms reported in the control group.

Table 7 summarized and compared the incidence of constipation, the most common AE for pain relief using analgesics, in the 2 study groups. No significant difference was found between the treatment and control group before analgesic treatment (*P* = .065). There were 25 cases of constipation (15 and 10 for mild and moderate level, respectively) in the control group after 7-day treatment, whereas there were only 9 cases of mild constipation in the treatment group. The difference of incidence of constipation after 1-week treatment was statistically significant between the control group and the treatment group (80.6%, 25/31 vs 29%, 9/31, *P* = .000). A total of 24 cases of constipation (7, 11 and 6 for mild, moderate and severe level, respectively) were found in the control group after 14-day treatment compared with only 1 case of mild constipation in the treatment group. The difference of incidence of constipation after 2-week treatment was statistically significant between the control group and the treatment group (80.6%, 24/31 vs 3.2%, 1/31, *P* = .000). These data suggested that CMV compress combined with analgesic treatment significantly reduce incidence and level of adverse events.

**Table 7:**
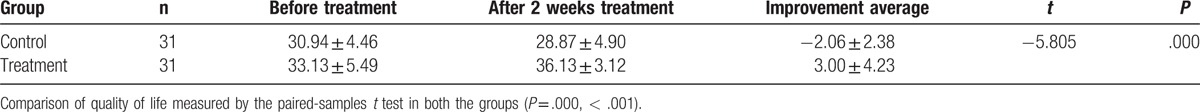
Comparison of the quality of Life (
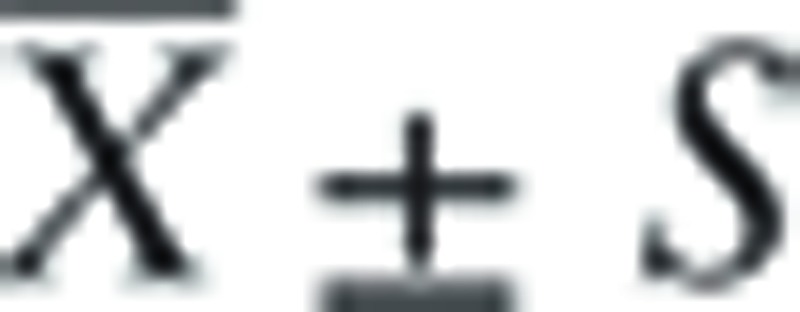
, score).

## 4 Discussion

Pain is a commonly reported symptom and a primary concern for patients with cancer. Although the etiology of cancer has not been completely illustrated, cancer pain relief can be achieved through various strategies, including the 3-step analgesic ladder management and biological and nerve block treatment. Approximately 50% of patients however, do not have their pain effectively controlled for various reasons.^[25]^ Regardless of the effectiveness of the 3-step analgesic ladder treatment, some patients fail to achieve pain relief due to intolerable side effects and the prevention of drug addiction.^[26]^ Recent research has reported that TCM treatment has good efficacy in alleviating cancer pain and improving the overall QOL of cancer patients.^[19]^ In the present study, cancer patients were treated with a 3-step analgesic ladder with or without CMWC, and the effect of the CMWC on pain relief, analgesic doses, adverse events and QOL of patients were assessed.

From the perspective of TCM, the etiology and mechanism of cancer pain involves a deficiency of healthy *qi*, invasion of pathogens (evil *qi*), obstruction of channels of healthy *qi* and blood stasis, which results in the accumulation of pain-producing chemicals. Therefore, the principles of TCM for cancer pain focus on strengthening healthy *qi,* expelling evil *qi* and activating and warming blood to treat both the symptoms and pathogens. Previous studies showed TCM together with the 3-step analgesic ladder achieved a total response rate of cancer pain relief greater than 80%.^[27,28]^ Our study also showed a significant difference in alleviating cancer pain using “*Wen Jing Zhi Tong Fang*” combined with the 3-step analgesic ladder treatment compared with the 3-step ladder alone (Fig. 1 and Table 8). The 4 constituents of the CMWC share common features as well as unique characters. Modern pharmacological research has reported that *Evodia rutaecarpa* (*Wuzhuyu*) can decrease blood pressure, improve the microcirculation, regulate body temperature and has anti-thrombotic, anti-allergic, anti-cancer, analgesic and anti-inflammatory effects.^[29]^*Semen sinapis* (*Baijiezi*) has anti-tussive, expectorant, anti-asthmatic, anti-radiation and anti-inflammatory effects^[30]^ and is attributive to the lung and stomach meridians. *Ephedra sinica* (*Mahuang*) can promote sweating, ventilate lungs to relieve dyspnea, and induce diuresis to disperse swelling.^[31]^ It is attributive to the lung, large intestine, and urinary bladder meridians. *Asarum sieboldii* (*Xixin*) is attributive to the lung and kidney meridians. It has been reported that Xinxin can expel heat, relieve pain and dyspnea, strengthen cardiac function, eliminate phlegm, improve the body's metabolism and immune system, and have anti-inflammatory and anti-convulsive effects.^[32]^ A combination of these herbs in appropriate proportions is expected to relieve cancer pain by stimulating healthy *qi*, dredging the meridians and collaterals, regulating *qi* and blood, and restoring the internal physiological functions of vital organs in the body.

**Table 8:**
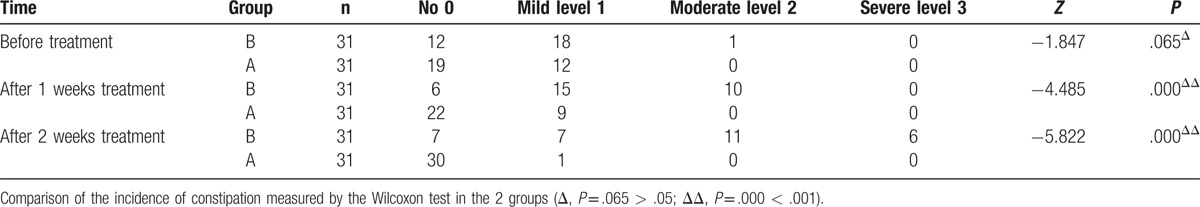
Comparison of the incidence of constipation in the 2 groups (n).

Previous studies have demonstrated the effectiveness of meridians in the back of regulating *qi* and blood flow, controlling the functions of internal organs (*zang fu*), improving the body's immune system and relieving pain.^[33–35]^ The back of the body is considered to be the region where *qi* from the individual internal organs converges and from where the *qi* and blood return to the targeted viscera. There are several important meridians located on the back of the body including: 1) the urinary bladder (UB) meridian on the first (*back shu*) and second line (*shu* point), which has close and broad connections with internal organs; 2) *Du* meridian, starting from the midpoint between the coccyx and the anus. It ascends along the spinal column of the body up to the top of the head and connects with the Ren meridian. The *Du* meridian enters the spine and brain and has associations with the kidneys, spine and brain. It also connects *UB*, *Yang Wei* and *Yang Qiao* meridians as well as several individual internal organs; 3) *Hua Tuo Jia Ji* acupoints located bilaterally in a row alongside the spine, is part of the CNS responsible for consciousness and has a close relationship with individual internal organs, limbs and connective tissues. In addition, the heated CMWC was deposited on the back meridian from T1-T12 vertebrae at the *Du*, *Back Shu* and *Jiaji* points. The role of the CMWC in pain relief was likely to be similar to that of ‘dermatomes’ associated with the T1-T12 vertebrae, which could activate the sympathetic nervous system and lead to autonomic neuropathy, resulting in disorders pertaining to internal organs, blood flow, alterations in nutrition supply and producing local symptoms in the case of thoracic vertebrae, spinal cord or nerve root damage. Conversely, a lesion of an internal organ could cause the local transmission of pain to the cerebral cortex and into spinal cord, resulting in referred pain.^[36,37]^ Therefore, the combined use of the 3-step analgesic ladder treatment with CMWC on the back meridians was expected to function in dredging *qi* of internal organs, regulating *yin/yang* and *qi/*blood, unblocking meridians/collaterals, resolving pain and easing the mind. Moreover, our study showed improved ORR of pain relief with the CMWC treatment in different locations (from 100% to 33.33% for upper and lower back pain, respectively, Fig. 2) compared with before therapy. Larger scale studies with a greater number of patients are needed to compare the effect of CMV for alleviating cancer pain in different locations.

Currently the WHO 3-step analgesic ladder strategy is the internationally recognized and widely accepted management strategy for cancer pain. However, various side effects related to non-opioid and opioid analgesics, such as nausea and vomiting, urinary retention, constipation, dizziness, respiratory inhibition and even coma, have been reported.^[24,38]^ Our study showed that only constipation was found in the treatment group (3-step analgesic ladder with CMWC treatment) and the incidence of constipation was significantly decreased in the treatment group compared with the control group (only 3-step analgesic treatment). Moreover, analgesic doses were significantly reduced after 1 week of CMWC treatment, suggesting the potentiality of using CMWC instead of western medicine in appropriate cancer patients to treat pain at an early stage of cancer. In agreement with other studies,^[19]^ our study showed improved QOL of cancer patients due to less side effects and decreased adverse events using CMWC combined with the 3-step analgesic ladder for cancer pain treatment.

A limitation of the study was that although the application of CMWCs on back meridians combined with WHO 3-step analgesic ladder treatment was effective in relieving cancer-related pain with reduced doses, less adverse reactions, and improved QOL, the research findings of this study were limited by a small sample size and short duration. It should be noted that in our study the CHW “*Wen Jing Zhi Tong Fang*” compress deposited on back meridians was combined with the 3-step analgesic ladder treatment. Further study is needed to address whether “*Wen Jing Zhi Tong Fang*” alone is effective in relieving cancer pain in appropriate cancer patients.

## 5 Conclusions

The current study demonstrated that “*Wen Jing Zhi Tong Fang*”, a CMWC, was effective in alleviating cancer pain, reduced side effects of adjuvant analgesics and improved QOL of cancer patients in combination with the WHO 3-step analgesic ladder treatment.
